# Optimizing and benchmarking *de novo* transcriptome sequencing: from library preparation to assembly evaluation

**DOI:** 10.1186/s12864-015-2007-1

**Published:** 2015-11-18

**Authors:** Yuichiro Hara, Kaori Tatsumi, Michio Yoshida, Eriko Kajikawa, Hiroshi Kiyonari, Shigehiro Kuraku

**Affiliations:** Phyloinformatics Unit, RIKEN Center for Life Science Technologies, 2-2-3 Minatojima-minami, Chuo-ku, Kobe, Hyogo 650-0047 Japan; Laboratory for Vertebrate Body Plan, RIKEN Center for Developmental Biology, 2-2-3 Minatojima-minami, Chuo-ku, Kobe, Hyogo 650-0047 Japan; Animal Resource Development Unit, RIKEN Center for Life Science Technologies, 2-2-3 Minatojima-minami, Chuo-ku, Kobe, Hyogo 650-0047 Japan; Genetic Engineering Team, RIKEN Center for Life Science Technologies, 2-2-3 Minatojima-minami, Chuo-ku, Kobe, Hyogo 650-0047 Japan

**Keywords:** RNA-seq, Transcriptome sequencing, *de novo* assembly, Completeness assessment, Library insert length, CVG (core vertebrate genes), Madagascar ground gecko

## Abstract

**Background:**

RNA-seq enables gene expression profiling in selected spatiotemporal windows and yields massive sequence information with relatively low cost and time investment, even for non-model species. However, there remains a large room for optimizing its workflow, in order to take full advantage of continuously developing sequencing capacity.

**Method:**

Transcriptome sequencing for three embryonic stages of Madagascar ground gecko (*Paroedura picta*) was performed with the Illumina platform. The output reads were assembled de novo for reconstructing transcript sequences. In order to evaluate the completeness of transcriptome assemblies, we prepared a reference gene set consisting of vertebrate one-to-one orthologs.

**Result:**

To take advantage of increased read length of >150 nt, we demonstrated shortened RNA fragmentation time, which resulted in a dramatic shift of insert size distribution. To evaluate products of multiple *de novo* assembly runs incorporating reads with different RNA sources, read lengths, and insert sizes, we introduce a new reference gene set, core vertebrate genes (CVG), consisting of 233 genes that are shared as one-to-one orthologs by all vertebrate genomes examined (29 species)., The completeness assessment performed by the computational pipelines CEGMA and BUSCO referring to CVG, demonstrated higher accuracy and resolution than with the gene set previously established for this purpose. As a result of the assessment with CVG, we have derived the most comprehensive transcript sequence set of the Madagascar ground gecko by means of assembling individual libraries followed by clustering the assembled sequences based on their overall similarities.

**Conclusion:**

Our results provide several insights into optimizing *de novo* RNA-seq workflow, including the coordination between library insert size and read length, which manifested in improved connectivity of assemblies. The approach and assembly assessment with CVG demonstrated here would be applicable to transcriptome analysis of other species as well as whole genome analyses.

**Electronic supplementary material:**

The online version of this article (doi:10.1186/s12864-015-2007-1) contains supplementary material, which is available to authorized users.

## Background

Transcriptome sequencing (RNA-seq) has become a standard strategy to capture the spatiotemporal expression of a genome. It has been applied to diverse organisms including those with limited prior sequence information, usually denoted as ‘non-model’ species [1–3]. RNA-seq targets transcribed regions that account for a minor fraction of whole genomes, at least in metazoans [4]. This compactness enables economical and rapid processing of sequencing and data analysis, which could be further improved via the optimization of various of parameters present in sample preparation, *de novo* short read assembly, and assembly product evaluation.

Modern high-throughput sequencers provide diverse sequencing modes with variable read lengths, read types (single read or paired-end read), and data sizes per run. Obviously, the choice of which sequencing mode to use influences the coverage of the transcriptome in *de novo* sequencing projects targeting sequence discovery, as well as influencing expression profiling in differential gene expression analyses. However, sample preparation protocols for many existing commercial kits do not provide practical instructions about their suitability for individual purposes and sequencing modes. For RNA-seq library preparation, there are few that introduce a choice of insert lengths with variable conditions for RNA fragmentation. For example, the standard protocol for Illumina TruSeq RNA Sample Prep Kit recommends intensive RNA fragmentation, which results in a high proportion of library molecules with the middle of their inserts sequenced from both ends. To maximize the potential of obtaining longer reads, it is preferable to prepare libraries with longer inserts using moderate RNA fragmentation.

Several computational programs employing short reads have been developed for producing *de novo* transcriptome assemblies [5–8]. Typical challenges in *de novo* transcriptome assembly include large variation of expression levels among transcripts, sequencing bias, and alternative splicing [6]. Merging multiple assemblies based on different k-mer lengths is an effective way of improving transcriptome assemblies because each transcript has different degrees of abundances [5, 9]. Thus, many of the transcriptome assemblers implement the multiple k-mer approach. On the other hand, Trinity, one of the most widely used transcriptome assemblers, allows only a fixed k-mer value (k = 25) when it is employed as a full program package [6]. So far, both Trinity and the multiple k-mer approaches have provided reasonable assembly results [10, 11]. *De novo* transcriptome assemblies are sometimes used as references to which short reads are mapped when transcriptome profiles are compared between multiple samples [12–14]. In such differential expression analyses, the mapping target, usually called the ‘reference’ assembly, is made from short reads from multiple sample sources, which requires a process that merges the sequences into one assembly. This merging can be hindered by among-sample variation of expression levels of individual genes and genetic backgrounds. To cope with these difficulties, it is worthwhile to analyze multiple methods for merging assemblies, provided that the merged assemblies are compared and evaluated on reasonable grounds (see [11]).

Evaluating *de novo* assembly products requires a multi-faceted assessment [15]. N50 length, a weighted median of assembly sequence lengths, is a widely used metric but does not give any clue about the completeness of the contents of the assembly, such as protein-coding genes. This aspect of assembly evaluation could be satisfied through the use of the pipeline CEGMA (Core Eukaryotic Genes Mapping Approach) [16, 17]. CEGMA makes use of 458 core eukaryotic genes (CEGs), with each gene consisting of orthologs that are conserved among six eukaryotic species (*Arabidopsis thaliana*, *Saccharomyces cerevisiae*, *Schizosaccharomyces pombe*, *Caenorhabditis elegans*, *Drosophila melanogaster*, and *Homo sapiens*), and reports the coverage of protein-coding genes in a particular set of assembled sequences [16]. Intuitively, executing CEGMA referring to a rigorously selected 248 gene subset of the 458 CEGs, which is composed of conserved genes with no or minimal paralog(s) from, is expected to yield accurate completeness assessment [17]. In reality, however, our preliminary analysis has shown that some CEGs have paralogs potentially misidentified as orthologs.

**Fig. 1:**
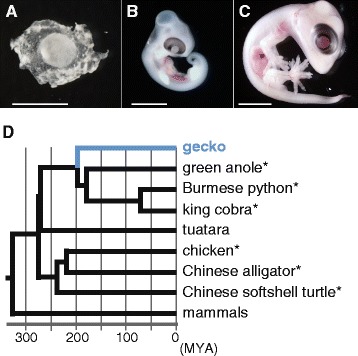
Animals used in this study. **a** Embryo of Madagascar ground gecko at four days before estimated date of oviposition (−4 days post oviposition, −4 dpo). **b** 9 dpo embryo. **c** 30 dpo embryo. Scale bars, 2 mm. **d** Molecular phylogenetic relationship between the gecko and other amniotes. Asterisks indicate the sauropsid species for which whole genome sequences have been published. Divergence times were based on the TimeTree project [22]

In this study, we reconstructed embryonic transcriptomes of the Madagascar ground gecko (*Paroedura picta*) (Fig. 1a). This species has a variety of benefits for use in developmental biology, including the availability of an elaborate embryonic staging system, feasibility of *in ovo* operational experiments, and non-seasonal high reproductivity [18–21]. In the reptilian order Squamata, large-scale sequence information is publicly available for anole lizard, Burmese python, and king cobra. Within Squamata, the lineage leading to Gekkonidae, to which the Madagascar ground gecko belongs, diverged from the lineage containing the above mentioned species approximately 200 million years ago (Fig. 1b) [22]. The phylogenetic position emphasizes the importance of producing sequence information for this animal lineage.

For efficient data production, we introduced modifications to a standard library preparation protocol to increase insert length, and exerted paired-end reads whose lengths were 150 nucleotides (nt) or more [23]. Developing technology could allow us to obtain much longer reads. To take advantage of this anticipated improvement, it could be useful to explore the coordination of the library preparation and sequence read length, as demonstrated in this study. To evaluate transcriptome assemblies with higher accuracy and resolution, we performed a careful examination of molecular phylogenies of genes in 29 vertebrate genomes, which resulted in the new reference gene set we designated CVG.

## Results

### Large-size library inserts improve connectivity of transcriptome assembly

**Fig. 2:**
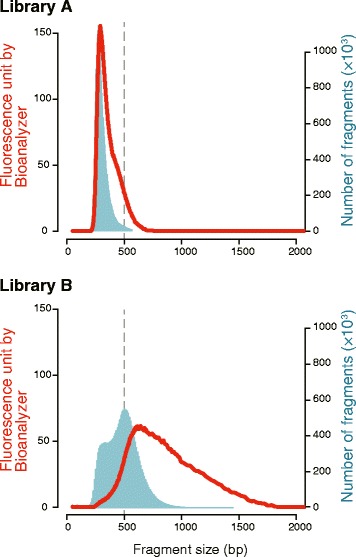
Size distribution of prepared and sequenced fragments. Fragment size distributions are shown for Library **a** and Library **b** (see Table 1). The red lines represent the size distributions reported by Agilent 2100 Bioanalyzer. The light blue areas represent inferred size distributions of the sequenced fragments. Insert sizes were extracted from the results of paired read mapping onto Assembly 1 (for Library **a**) and Assembly 2 (for Library **b**) (see Table 2 for details of these assemblies). A fragment size is a sum of the sizes of the insert and the TruSeq adapters

We introduced two major changes to the standard protocol of Illumina TruSeq RNA Sample Prep Kit in regards to RNA fragmentation and size selection of inserts. First, we shortened RNA fragmentation time to increase overall insert lengths. Second, in DNA purification, we decreased the volume of Agencourt AMPure XP, aiming to retain libraries with large-size fragments, e.g., those longer than 300 bp. Using total RNA extracted from a gecko embryo four days before the estimated date of oviposition [−4 days post oviposition (dpo)], we prepared libraries without and with the above modifications to the standard protocol (Library A and Library B, respectively; Table 1). For Library A and B, we loaded the same number of DNA molecules, based on prior quantification, in the same numbers of lanes (Table 1), and confirmed that it resulted in comparable numbers of reads (Additional file 1). Mapping of the reads to the *de novo* assemblies (see Methods) indicated that Library B had larger overall fragment sizes (665 bp on average) and a broad size distribution compared to Library A (349 bp on average; Fig. 2). Additionally, the fraction corresponding to reads shorter than 300 bp was largely reduced in the size distribution of Library B.

**Table 1:** Properties of RNA-seq libraries

Library	Library preparation				Sequencing
	RNA source	Duration of RNA fragmentation (min)	× AMPure volume (targeted fraction to retain)	PCR cycles	
A	-4 dpo whole embryo^a^	8	× 1.6 (>100 bp)	6	HiSeq 1/4 lanes, 171 cycles paired-end
B		2	× 0.7 (>300 bp)	6	
C				6	MiSeq 1/4 lanes, 250 cycles paired-end
D	9 dpo whole embryo	8	× 1.6 (>100 bp)	2	HiSeq 1/4 lanes, 171 cycles paired-end
E		2	× 0.7 (>300 bp)	4	
F	30 dpo head	4	× 1.0 (>150 bp)	6	HiSeq 2/3 lanes, 151 cycles paired-end
G	30 dpo liver			6	
H	30 dpo tail			6	

^a^Embryo of 4 days before the estimated day of oviposition

In order to evaluate the performance of the protocol modifications, we sequenced the libraries on the Illumina HiSeq with 171 cycles [23]. It is expected that longer reads should increase the proportion of large-size fragments, for which paired reads will cover the full stretch. We inferred the size of the sequenced inserts by mapping the paired reads to transcript contigs that were assembled from the obtained reads (see Methods). The result showed that the sizes of the sequenced fragments were smaller than the sizes of the majority of the fragments in the prepared library (Fig. 2). Nevertheless, the average size of sequenced fragments of Library B (360 bp) was larger than that of Library A (180 bp) (Fig. 2), which was consistent with the observations of the average sizes of the fragments in the prepared library.

In order to examine whether large-size inserts improve assembly, we compared N50 lengths between Assembly 1 and Assembly 2, which were made from the sequenced reads of Libraries A and B, respectively (Additional file 1). After adapter trimming and quality filtering (see Methods), Library A still had more sequence reads (22.7 million pairs composed of 6.87 Gbp) than Library B (21.2 million pairs composed of 6.79 Gbp). In comparison, Assembly 2 had larger N50 length than Assembly 1 (Table 2), in spite of its smaller read number. The improvement of N50 length in Assembly 2 mainly resulted from the absence of short contigs (<500 bp) (Additional file 2). A comparison of N50 length based on only one representative contig per gene (‘subcomponent’ in Trinity), instead of all contigs, also demonstrated the superiority of Assembly 2 (Additional file 1). These results indicate that our modification to the library preparation protocol has the potential to produce sequence reads that can be assembled into longer contigs. This tendency in assembly connectivity was also confirmed in another trial using different RNA sources (Table 2; Additional files 1, 2, and 3).

**Table 2:** Transcriptome assembly statistics

Assembly No.	Individual^a^ or integrated^b^ assembly	Assembly approach	Number of fragments (×10^6^) ^c^	Raw assembly		Assembly filtered by mapping count (≥5)		N50 length (bp)
				Number of contigs	Number of subcomponents	Number of contigs	Number of subcomponents	
1	A	Trinity	22.719	222178	168924	106323	62636	3091
2	B		21.224	228165	159338	94371	45267	3634
3	C		3.569	104985	83417	37504	22331	3093
4	D		23.712	417291	291424	204328	104294	3693
5	E		16.037	383737	246347	149926	56669	4149
6	F		75.929	798982	562528	358433	182611	3956
7	G		82.453	787608	541906	375297	191055	3860
8	H		81.033	525154	348570	250433	115476	4090
9	Integrated	All-in-one by Trinity	326.676	1214573	852257	653132	387456	2680
10		All-in-one by SOAPdenovo-trans, multiple k-mer lengths	326.676	1087900	745363	748019	422329	4854
11		Assembly following Trinity’s normalization	39.593^d^	1465425	721986	972512	330937	3755
12		Assembly after khmer	33.251^d^	1464412	741241	945799	314023	2898
13		Assembly and clustering	326.676	1562282	939252	996336	457323	3897

^a^Corresponding library symbols (see Table 1) are included for individual assemblies

^b^Integration of all the individual assemblies

^c^Number of fragments for which both of the pairs passed quality control

^d^Note that this is a number of fragments after *in silico* normalization

### Derivation of new reference gene set for vertebrates

Coverage of the protein-coding landscape, which we call ‘completeness’ in this article, is one of the typical quality measures of *de novo* transcriptome assembly [13, 24]. The pipeline CEGMA has been used for this purpose in eukaryotes [16, 17]. A subset of 458 CEGs possessing no or minimal paralogs (248 CEGs) is used as default in completeness assessment by CEGMA [17]. Each of the CEGs consists of only one gene per species, even if the species has its paralog due to a lineage-specific duplication. To examine potential effects of such additional paralogs, we analyzed molecular phylogenies of all the 248 CEGs based on the gene trees provided in Ensembl [25]. Overall, 71 duplication events in 64 CEGs, out of the 248 CEGs, were revealed to have occurred in the lineage leading to vertebrates, and were dated at different evolutionary periods by referring to Ensembl Compara [25] (Additional file 4: Figure S3A). These paralogs could potentially cause substantial overestimation of completeness in vertebrate transcriptome or genome sequences. One of the CEGs prone to such effect, the glucose-6-phosphate dehydrogenase (G6PD) gene, is shown as an example in Additional file 4: Figure S3B. Although the latest chicken genome assembly harbors no G6PD ortholog, CEGMA misidentified its ancient duplicate, hexose-6-phosphate dehydrogenase (H6PD), as a G6PD ortholog in the chicken genome. These caveats demonstrate the need of a carefully validated gene set customized for vertebrates to improve the accuracy of completeness assessment.

**Fig. 3:**
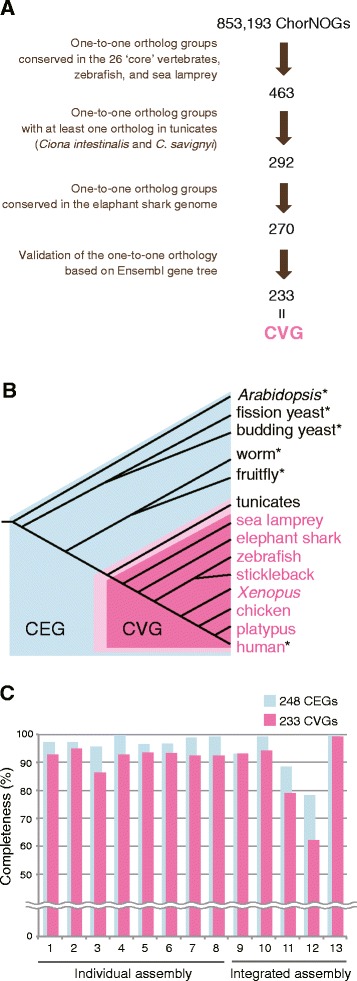
Core Vertebrate Genes (CVG). **a** Flowchart showing selection procedure of the CVG from the chordate ortholog groups of eggNOG v4.0 (ChorNOGs). The 26 core species were specified by the eggNOG. Components of the CVG were shown in Additional file 5. **b** Taxonomic ranges of CEG (on a light blue background) and CVG (on a magenta background). The CEG consists of the six the species with asterisks, and the CVG set for CEGMA consists of the eight species in magenta. Tunicate orthologs were used as outgroup in order to distinguish one-to-one orthologs conserved in vertebrates from those with additional paralogs duplicated in the vertebrate lineage. Those with no additional vertebrate paralog were included in CVG. **c** Completeness scores of the transcriptome assemblies assessed by CEGMA referring to the 248 CEGs and 233 CVGs. The scores indicate proportions of the genes recognized as ‘complete’ in individual assemblies by CEGMA out of 248 CEGs and 233 CVGs. See Additional file 8 for the results of an equivalent assessment with BUSCO

Instead of using the 248 CEGs, we adopted 233 core vertebrate genes (CVGs; Fig. 3a; Additional file 5). Each of the CVGs is composed of one-to-one orthologs based on eggNOG of 29 species including jawed vertebrates as well as cartilaginous fish and cyclostomes [26] (Fig. 3a; see Methods). All of the 233 CVGs were required to have tunicate orthologs, in addition to having the one-to-one orthologies validated by another ortholog database, Ensembl Compara. These conditions ensured that the one-to-one orthology was retained throughout vertebrate evolution (Fig. 3a), despite large-scale gene (or genome) duplication events in early vertebrates. To consolidate this gene set for CEGMA, eight vertebrate species were selected from the CVG set (Fig. 3b; also Additional file 5): human (*Homo sapiens*), platypus (*Ornithorhynchus anatinus*), chicken (*Gallus gallus*), *Xenopus tropicalis*, zebrafish (*Danio rerio*), stickleback (*Gasterosteus aculeatus*), elephant shark (*Callorhinchus milii*), and sea lamprey (*Petromyzon marinus*). Out of the 233 CVGs, only 17 are also included in the 248 CEGs (Additional file 5), and thus there is a substantial difference between the compositions of CVG and CEG. On the other hand, the proportion of the genes identified as ‘complete’ by CEGMA (completeness score) was positively correlated between the CEG and CVG sets (Fig. 3c; R = 0.95; *p* = 1.0 × 10^−6^). Generally, CEGMA referring to the CVG yielded lower completeness scores than with the CEG, and the completeness scores with the CVG showed a higher variation than with the CEG (Fig. 3c). These results indicate that the CVG set has enabled completeness assessment with fewer false positives and higher resolution.

### Assessment of assemblies reconstructed from multiple samples

**Fig. 4:**
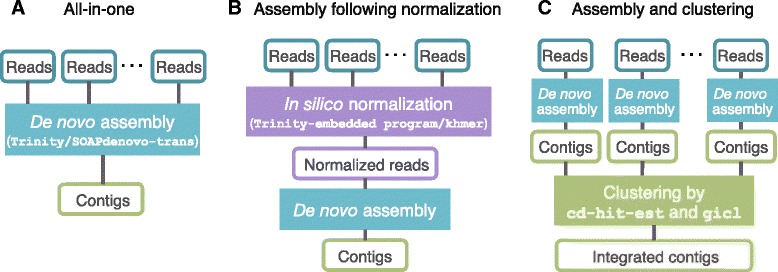
Demonstrated assembly approaches. **a** All-in-one assembly using either Trinity or SOAPdenovo-trans resulted in Assembly 9 and Assembly 10, respectively). Assemblies employing multiple k-mer lengths based on SOAPdenovo-trans were merged by the same procedure as that of merging individual assemblies for the assembly and clustering approach below. **b** Assembly following *in silico* normalization of short reads with the normalization function implemented in Trinity and khmer resulted in Assembly 11 and Assembly 12. **c** Clustering following assembly was performed with both cd-hit-est and gicl (Assembly 13). See Table 2 for statistics of the generated assemblies and Methods for the details of these three individual procedures

We sequenced eight RNA-seq libraries using total RNA extracted from three embryos at different stages (Fig. 1a; Tables 1 and 2). Because their expression profiles and genetic backgrounds were thought to vary across the samples, we demonstrated several approaches to integrating individual assemblies derived from all of the samples (Fig. 4). All-in-one approach assembles reads from multiple samples at a time (Fig. 4a). *In silico* normalization removes redundant reads prior to the all-in-one assembly (Fig. 4b), which should lead to reduced computational costs [13, 27]. Another approach is assembly and clustering: assemblies from individual samples are clustered based on sequence similarity (Fig. 4c). We examined five approaches and evaluated their products (See Methods; Assembly 9-13 in Table 2). According to the completeness assessment by CEGMA, the completeness scores of the integrated assemblies varied substantially even though these assemblies were produced from the same set of sequence reads (Fig. 3c and Additional file 6). An integrated assembly is expected to have a completeness score equal to or larger than those of any individual assemblies. This expectation was satisfied only with Assembly 13, which was built with the assembly and clustering approach (Fig. 3c). Assembly 10, based on the all-in-one approach by SOAPdenovo-trans employing multiple k-mer lengths, showed the second largest score. For this assembly however, the completeness score with the CEG did not exceed that of the individual assemblies, whereas it did exceed them with the CVG (Fig. 3c and Additional file 6).

Assembly following *in silico* normalization can be an alternative approach when sequence data is too large to handle with available computational resources (e.g., more than 200 million reads) [13, 27]. *In silico* normalization reduced redundancy from the input sequence reads and dramatically decreased the data size down to approximately 10 % (Table 2). However, the completeness scores of Assembly 11 and Assembly 12 were much poorer than even that of Assembly 9, the all-in-one assembly by Trinity.

In order to evaluate the proportion of short read pairs that properly participated in an assembly, we mapped the reads to the assembled contigs and calculated the proportions of the pairs with the ‘properly-paired’ flag in the mapping results (Additional file 7). The result showed that Assembly 10 had the highest mapping rate among the integrated assemblies. In addition, Assembly 10 also exhibited the largest N50 length among all the integrated assemblies, supporting its high overall connectivity (Table 2). Assembly 13 showed the second largest mapping rate and N50 length (Table 2 and Additional file 7). While the qualities of Assembly 10 and Assembly 13 were comparable to each other, we finally chose Assemble 13 as the transcript sequence set representing this study because of its higher completeness score than any other individual assembly. Assembly 13 was composed of 996,336 contigs and 457,323 subcomponents, of which 444,832 were homologous to annotated protein-coding genes of vertebrate genomes that were selected for this purpose (E-value < 1E-10 based on BLASTX; see Methods).

### Adapting CVG to the newly introduced completeness assessment tool, BUSCO

The CVG is applicable as a core gene set to not only CEGMA, but widely to other complete assessment methods. We applied the CVG to BUSCO, a recently introduced tool [28] (See Methods). Using the 13 gecko transcriptome assemblies built in this study, we demonstrated that the completeness scores with BUSCO referring to the 233 CVGs were comparable to those with CEGMA, and Assembly 13 again showed the highest completeness score with BUSCO (Additional files 6 and 8).

Originally, BUSCO offers 3,023 ortholog groups of 41 vertebrates that retain one-to-one orthology in almost all of these species based on OrthoDB [29]. However, the BUSCO’s vertebrate gene set is composed of bony vertebrates only, which potentially could fail to identify orthologs of cartilaginous fish and cyclostomes. For example, using the Japanese lamprey (*Lethenteron japonicum*) genome assembly, BUSCO referring to its original vertebrate gene set showed a completeness score of only 21 %. On the other hand, the completeness scores of BUSCO referring to the metazoan gene set and the CVG (70 % and 73 %, respectively) were comparable to those based on CEGMA (83 % and 77 % referring to the CVG and CEG, respectively). Remarkably, execution of BUSCO referring to the CVG dramatically reduces computational time. Using the Assembly 13, a BUSCO run referring to the CVG is completed in approximately 15 min, while a BUSCO run referring to the vertebrate gene set and a CEGMA run referring to the CVG takes much longer (116 min and 438 min, respectively). We propose the CVG as a handy gene set to be used with BUSCO for completeness assessment of vertebrate genome and transcriptome assemblies.

## Discussion

### Coordination between library insert size and read length

In this study, we modified the library preparation protocol for RNA-seq on the Illumina platform to keep up with further developing sequencers. Although the protocol modifications increased the connectivity of the transcriptome assemblies, there still remains room for further improvement. The fragment size distribution of the library prepared with modifications shifted towards the fraction of small-sized fragments (Fig. 2). This shift was notable for the size distribution of Library B: the average size of the actually sequenced fragments was more than 300 bp shorter than that of the fragments in the prepared libraries. This shift of the size distribution was observed regardless of the choice of sequencer models (Fig. 2; Additional file 2). One possible explanation for this is insufficient size selection with AMPure beads. Although its volume was reduced to retain only fragments longer than 300 bp, those shorter than that may not have been sufficiently removed (Fig. 2). The other possible explanation is that ‘clusters’ in Illumina chemistry might not be generated evenly on a flow cell because of variable fragment lengths, although the total size of the output does not differ among sequencer runs for libraries with different fragment length distributions. The Rapid SBS kit v2, which has been released recently, improves robustness against high cluster density through the use of an upgraded HiSeq Control Software (HCS).

### Improved completeness assessment based on the 233 CVGs

CEGMA has been a standard for assessing genome assembly and is now widely used for assessment of transcriptome assemblies [30]. However, the presence of the paralogs in the 248 CEGs potentially results in overestimation of the completeness assessment. To improve the accuracy and resolution of the completeness assessment of the gecko transcriptome assemblies, we derived a new gene set composed of one-to-one orthologs of vertebrates (CVG). More conservative completeness scores were computed with CEGMA referring to the 233 CVGs than to the 248 CEGs, suggesting more accurate assessment based on the former (Fig. 3c; Additional file 6). Indeed, none of the CEGMA executions on the 13 assemblies produced false-positive orthologs to the CVGs, as shown in the example of G6PD tree. In contrast, each assessment based on the CEG had approximately 2–5 % of such false positives. False positives in completeness assessment are considered to be more problematic in evaluating transcriptome assemblies than genome assemblies. This is because an absence of a particular transcript in transcriptome assemblies can be caused by no or extremely low gene expression in addition to insufficient sequence read numbers. Establishing a set of one-to-one orthologs for a particular taxonomy group, as demonstrated in the present study, would be applicable to other taxonomic groups.

Members of a one-to-one ortholog group of a specific taxon consequently share high sequence similarity, leading to fewer false positives caused by ancient paralogs being misidentified as orthologs. CEGMA detects genes homologous to a given reference gene sequence and recognizes those satisfying the HMMER score cutoff for the given gene as orthologs. The 233 CVGs have higher HMMER score cutoffs (median, 151.8) for ortholog detection than the 248 CEGs (median, −68.76) since their members are widespread across eukaryotes (Fig. 3b). In addition, orthologs of longer sequences result in a complete assessment with higher resolution because contigs with more than 70 % coverage in length to the HMMER profiles are recognized as ‘complete’ by CEGMA [17]. The larger the lengths of the HMMER profiles are, the less likely the contigs satisfying the ‘completeness’ are reconstructed. Indeed, the HMMER profiles of the CVGs had larger length than those of the 248 CEGs (medians, 557 aa and 379 aa, respectively), leading to the higher resolution of the assessment based on CVG. This high resolution can also be achieved by high sequence similarity among members in a CVG because truncated sequences result in lower HMM scores than the given thresholds, leading to exclusion from ortholog candidates. It is noted that the completeness score of Assembly 13 based on the CVG almost reached 100 %, demonstrating that low completeness scores of the CVG (Fig. 3c) is not caused by the absence of the expression of orthologs in transcriptomes. This suggests that the set of the 233 CVGs better assesses the contents of embryonic transcriptomes of vertebrates, at least.

The new tool, BUSCO, will provide versatile solutions for completeness assessment at various levels. In our analysis, completeness assessment using BUSCO referring to the CVG, which also showed the highest completeness score for Assembly 13, performed similarly to the assessment using CEGMA (Additional files 6 and 8). BUSCO originally offers a vertebrate ortholog set (3,032 groups). This gene set generally performs with similar resolution in completeness assessment to CVG (Additional file 6). In addition, CVG and the BUSCO’s vertebrate ortholog set showed similar performances to identify orthologs in comprehensive transcript sequence sets of diverse bony vertebrates (Additional file 9). Running BUSCO using CVG as a reference has two advantages. The first is that the CVG set includes a cartilaginous fish and a cyclostome, providing a wide taxonomic range for completeness assessment of high sensitivity. Secondly, the fewer components of the CVG allow a much more rapid computation by BUSCO. For public uses, we included the BUSCO-compatible CVG data set in the CVG suite.

### Transcriptome assembly of Madagascar ground gecko

Among the five integrated assemblies, Assembly 13 and 10 showed markedly high completeness, connectivity, and accuracy (Fig. 3c; Table 2; Additional files 6 and 7). On the other hand, it was demonstrated that the other assemblies failed to reconstruct considerable numbers of transcripts, which were retained in the individual assemblies (Fig. 3c). Assembly 13 was a merger of the individual assemblies, while Assembly 10 was one of the all-in-one assemblies with different k-mer lengths. Employing multiple k-mer lengths is advantageous in transcriptome assembly because different k-mer lengths show different effective ranges of sequence depths for transcriptome assembly and cover different parts of a transcriptome [5, 9]. The superiority of clustering of individual assemblies to all-in-one assemblies can be explained by variation of expression profiles among samples. This variation of sequence depths for a transcript should increase the possibility of reconstructing a full-length transcript sequence, even based on a single k-mer length.

In general, when typical *de novo* transcriptome assembly programs are used, the number of resultant contigs constantly increases along with the number of reads fed into the program [30] (Table 2). One possible reason for this is the abundance of so-called ‘leaky’ transcripts from intergenic regions [31] and unprocessed mRNA [32] in addition to contaminated genomic DNA [13]. Such molecules can be contained in the starting materials of library preparation, which will eventually result in low counts of reads after sequencing. To combat this, we excluded contigs with mapping counts of fewer than five in the assembly procedure (see Methods), which reduced the number of contigs of Assembly 13 down to 64 % (Additional file 1). In addition, N50 length of the filtered assembly increased by 336 bp compared with that of Assembly 13. Importantly, the completeness scores of this filtered assembly remained unchanged, suggesting that contigs with low mapping counts rarely include sequences derived from protein-coding genes conserved across vertebrate species. Indeed, in Assembly 13, only 5.6 % of the contigs homologous to the vertebrate genes were included in the discarded contigs. The representative assembly, Assembly 13, is available in the online gateway Reptiliomix linked from our laboratory website (http://www2.clst.riken.jp/phylo/reptiliomix.html), allowing data downloading and similarity searches.

## Conclusions

Our modified library preparation protocol for RNA-seq increased the sequence insert sizes, which adapts to the recent upgrades of sequence read length in high throughput sequencers. In addition, this protocol improved connectivity of *de novo* transcriptome assemblies. Our study showed that assembly integration based on the assembly and clustering approach by Trinity and the all-in-one approach based on multiple k-mer lengths by SOAPdenovo-trans produced assemblies of high quality. Because the degree of variations in expression levels and genetic backgrounds differs in each experiment, it is useful to assess multiple approaches of assembly integration. If a computational resource is limited, the assembly and clustering approach will be reasonable: it takes comparable computational time to the multiple k-mer lengths by SOAPdenovo-trans but requires less memory space. Lineage-specific one-to-one ortholog sets will be of help for performing completeness assessment in a uniform framework across diverse species. Our approaches to library preparation and assembly completeness evaluation would also be applicable to *de novo* genome assemblies.

## Methods

### Library preparation and sequencing

All animal experiments and housing were conducted in accordance with guidelines approved by the RIKEN Animal Experiments Committee (Approval IDs AH25-05-1 and AH14-05-47). Embryos of the Madagascar ground gecko, *Paroedura picta*, were provided by Animal Resource Development Unit, RIKEN CLST. Eggs of 9 and 30 dpo were collected from natural ovipositions, respectively, and an egg of four days before the estimated date of oviposition [−4 days post oviposition (dpo)] was extracted from a egg-bearing female. Total RNA was extracted using TRIzol reagent (Life Technologies), and RNA-seq libraries for non-stranded paired ends were prepared by Illumina TruSeq Total RNA Sample Prep Kit according to its standard protocol unless specifically described below. After confirming the reproducibility in a different species (data not shown), we shortened RNA fragmentation time from eight to two (Library B, C, and E) or four (Library F—H) minutes. In DNA purification, we applied x0.7 (Library B, C, and E) or x1.0 (Library B, C, and E) volume of Agencourt AMPure XP, instead of x1.6. These conditions were summarized in Table 1. The libraries were sequenced with HiSeq 1500 (Illumina inc.) operated by HiSeq Control Software v2.0.12.0 using Rapid SBS kit v1 as well as MiSeq using MiSeq Reagent kit v2, in the read lengths designated in Table 1.

### Quality control of sequenced reads

Raw nucleotide bases were called with RTA 1.17.21.3 and converted to the Fastq-format files with bcl2fastq 1.8.3 (Illumina Inc.). The short reads were deposited in the DDBJ Short Read Archive (DRA) database under the accession numbers PRJDB4004. Initially, qualities of sequence reads were checked with FastQC v0.10.1 (http://www.bioinformatics.babraham.ac.uk/projects/fastqc/), and no marked abnormalities were observed for all of the samples. Adapter sequences and low quality bases (<Q30) were trimmed from the 3′-ends by trim_galore (http://www.bioinformatics.babraham.ac.uk/projects/trim_galore/), in which cutadapt is implemented [33], discarding the reads of shorter than 50 bp after adapter and quality trimming. Low quality reads in which proportion of the bases ≥ Q30 was less than 80 % were discarded by the program fastq_quality_filter in FASTX Toolkit 0.0.13 (http://hannonlab.cshl.edu/fastx_toolkit/index.html). Fragments retaining paired reads were used for *de novo* assembly.

### *De novo* assembly

The assemblies for individual libraries (Assembly 1–8 in Table 2) were built by Trinity r20131110 [6] which employs a single k-mer length (k = 25). The two all-in-one assemblies were built using the reads of all the libraries by Trinity (Assembly 9) and SOAPdenovo-trans v1.03 [8] based on multiple k-mer lengths (k = 21, 25, 31, 41, 51, 61, 71, 81, and 91) (Assembly 10). For Trinity, we employed default parameters except the ‘group_pairs_distance’ option setting at 1000 for assembling the fragments with long inserts (Assembly 2, 3, 5, 6, 7, 8, 11, and 12). For SOAPdenovo-trans, we set parameters as follows: max_rd_len = 250, rd_len_cutof = 250, avg_ins = 300, reverse_seq = 0, asm_flags = 3, and map_len = 32. The contigs shorter than 200 bp were discarded from Assembly 10, as the default setting of Trinity. In order to remove redundancy of multiple sequences derived presumably from identical transcripts, contigs assembled by SOAPdenovo-trans with multiple k-mer lengths were merged by cd-hit-est v4.6.1 [34] with the similarity threshold of 99 % and word size at eight nucleotides, followed by clustering using gicl v0.0.1 (http://sourceforge.net/projects/gicl/) [35] with the similarity threshold of 95 % and the overlap length threshold of 50 bp. *In silico* normalizations were performed using the reads of all the libraries by two different programs in order to produce sequences showing unimodal k-mer coverage distributions with averages around 25: khmer 0.2 with options of “-K 20 –C 20” [27] and normalize_by_kmer_coverage.pl implemented in Trinity with options of “--KMER_SIZE 25 --max_cov 50” [13]. The resultant reads from the *in silico* normalizations were assembled with Trinity into Assembly 11 and Assembly 12. The assemblies for the individual libraries were merged into Assembly 13, as performed for Assembly 10. For Assembly 10 and Assembly 13, which underwent a post-assembly merge, we reassigned gene-transcript relationships by performing single linkage clustering of the contigs derived from the same locus. In order to remove the contigs with minimal read depths that possibly resulted from so-called ‘leaky’ transcription [31], all assemblies were subjected to further modification as follows. The reads were mapped to the contigs in each assembly with Bowtie2 version 2.2.2 end-to-end mode [36]. Using the mapping results, we excluded from the assemblies the contigs on which fewer than five reads were mapped, based on read counts with eXpress v1.5.1 [37]. The mapping and read count were processed using the wrapper align_and_estimate_abundance.pl in Trinity with default parameters except the ‘max_ins_size’ option set at 1500 for Assembly 2-3, and 5-13: the option corresponds to the ‘maxin’ option of Bowtie2 and is set at 800 as default in the wrapper.

In order to confirm that the discarded contigs contain little substantial information for transcriptome analyses, those contig sequences were subjected to searches for possible protein-coding regions with homologs in annotated vertebrate protein databases. For this purpose, similarity searches were carried out using BLASTX [38] in protein sequences of 13 vertebrates with an E-value cutoff of 1E-10: human (*Homo sapiens*), dog (*Canis familiaris*), opossum (*Monodelphis domestica*), chicken (*Gallus gallus*), zebra finch (*Taeniopygia guttata*), Chinese alligator (*Alligator sinensis*), Chinese softshell turtle (*Pelodiscus sinensis*), green sea turtle (*Chelonia mydas*), green anole (*Anolis carolinensis*), Burmese python (*Python molurus bivittatus*), *Xenopus tropicalis*, stickleback (*Gasterosteus aculeatus*), and elephant shark (*Callorhinchus milii*). Protein sequences of Chinese alligator and green sea turtle were obtained from GigaDB, and those of Burmese python and elephant shark were obtained from NCBI Genbank and Elephant Shark Genome Project web page (http://esharkgenome.imcb.a-star.edu.sg/), respectively. Protein sequences of the other species were downloaded from Ensembl release 75.

### Selection of components of CVG

The core vertebrate genes (CVG) were defined as one-to-one orthologs selected based on eggNOG [26] as follows. Initially, we extracted 463 chordate ortholog groups of eggNOG v4.0 (ChorNOGs) [26] that were composed of one-to-one genes of the 26 ‘core’ vertebrates defined by eggNOG, zebrafish (*Danio rerio*), and sea lamprey (*Petromyzon marinus*). From them, 292 groups possessing at least one ortholog of either *Ciona intestinalis* or *C. savignyi* were selected. Orthologs of elephant shark, whose genome assembly was released later, were added to the gene sets based on the BLASTP [38] reciprocal best-hit approach, and one-to-one elephant shark orthologs were identified in 270 groups out of the 292. The one-to-one orthology of 233 gene sets were systematically validated by gene trees produced by Ensembl release 70 [25], and examined with manual curation when necessary (also see Fig. 3a). Finally we extracted the one-to-one orthologs of eight species, human, platypus (*Ornithorhynchus anatinus*), chicken, *Xenopus tropicalis*, zebrafish (*Danio rerio*), stickleback, elephant shark and sea lamprey from each of the gene sets and defined the selected gene set as 233 CVGs for CEGMA (Additional file 4).

### Assessment of assembly

N50 lengths were computed by TrinityStats.pl embedded in Trinity [13]. Short reads were mapped to contigs by Bowtie2 [36], and mapping rates were obtained from its summary output. For completeness assessment, we applied CEGMA version 2.4 [17] based on the 248 CEGs and 233 CVGs separately. Using the CVG gene set for CEGMA, containing the eight vertebrates, HMMER profiles [39] were generated by HMMER 3.0 based on the multiple amino acid alignments processed by MAFFT v7.158b [40] following format conversion into HMMER 2.X. The HMMER bit score cutoffs of the CVGs were computed according to the criterion proposed by Parra et al. [16, 17]: the cutoff values for the standard CEGMA and the completeness analysis, corresponding to the ‘profiles_cutoff.tbl’ and ‘completeness_cutoff.tbl’ files in the original CEGMA package, respectively. We computed the cutoff values of the completeness analysis as maximum hmmsearch bit scores between the HMMER profiles and proteins from any transcripts of the eight species instead of the proteins from the representative transcripts of the genes. In order to conduct the complete assessment using custom gene sets, we modified the scripts of ‘cegma’ and ‘completeness’ implemented in the CEGMA.

We identified false positives in the CEGMA results for the gecko transcriptome assemblies based on a BLASTP search [38]. Using a gecko protein that was predicted as an ortholog of a CVG/CEG by CEGMA, we searched for its best-hit homolog in the human proteins. If this human best-hit was a paralog to the human protein of the CVG/CEG and if these paralogs were duplicated before the split of mammals and sauropsids, the protein predicted by CEGMA was recognized as a misidentified ortholog to the gene group. In this analysis, the human protein sequences and inferred timings of gene duplications were obtained from Ensembl release 70.

In addition to CEGMA, we conducted completeness assessment using BUSCO v1.1 [28] referring to the CVG, and the CVG dataset for BUSCO was prepared as follows. Using the whole sequence set (29 species) of the CVG, HMMER profiles [39] were generated by HMMER 3.1b2 based on the multiple amino acid alignments processed by MAFFT v7.158b [40]. Protein profiles of the CVG for Augustus were generated with msa2prfl.pl in Augustus 3.1 [41] based on the multiple alignments. The consensus sequence of each CVG was inferred by hmmemit in the HMMER. The cutoff values of sequence lengths were computed according to the criterion described previously [28]. As for cutoff values of HMMER bit scores, we used the values for CEGMA described above, instead of those according to the original criterion by BUSCO.

Mapping rates of the assemblies were computed by SAMtools version 0.1.19 [42] using the mapping files that were made for counting mapped reads to the contigs in the previous subsection. Insert lengths of the fragments were estimated with CollectInsertSizeMetrics in Picard Tools version 1.90 (http://broadinstitute.github.io/picard/). For this purpose, in mapping, we used first 50 nucleotides of each paired read so that paired-reads were not mapped overlapping each other. These reads were mapped to the assemblies using Bowtie2 with the same parameters described in the subsection “*De novo* assembly” in Methods.

### Molecular phylogenetic analysis

Peptide sequences of G6PD and H6PD were collected from the gene set of KOG0563 in CEGMA as well as NCBI Genbank and Ensembl release 70 with the assistance of aLeaves [43]. The homologous peptides were aligned with six different approaches: forward and reverse directions by MAFFT v7.158b [40], Clustal Omega 1.2.0 [44], and T-Coffee 10.00.r1613A [45]. The consensus multiple alignment from the six procedures was made by M-Coffee [46] implemented in the T-Coffee package. Unambiguous alignment sites were selected based on trimAl version 1.4 with the automated1 option following removal of gapped sites. Molecular phylogenetic trees were reconstructed based on RAxML version 7.5.7 [47] assuming the PROTCATWAG model with 1,000 bootstrap replicates ("−f a" option) and PhyloBayes 3.3f assuming the CAT-GTR model [48].

## Availability of supporting data

The raw reads are available at DRA under the BioProjet ID PRJDB4004. The transcriptome assembly, the CVG datasets for CEGMA and BUSCO, and the extended CEGMA scripts are available at Reptiliomix and our laboratory web site (http://www2.clst.riken.jp/phylo/reptiliomix.html). The other data sets supporting the results of this article are included within the article and its additional files.

## Additional files

Additional file 1: Table S1. Detailed assembly statistics based on numbers of detected genes. (PDF 61 kb) Additional file 2: Figure S1. Size distribution of contigs in assemblies. (PDF 80 kb) Additional file 3: Figure S2. Size distribution of prepared and sequenced fragments. (PDF 121 kb) Additional file 4: Figure S3. Paralogs in the CEGs. (PDF 95 kb) Additional file 5: Table S2. Components of the 233 CVGs. (XLSX 132 kb) Additional file 6: Table S3. Completeness scores of transcriptome assemblies based on numbers of detected genes. (PDF 62 kb) Additional file 7: Figure S4. Proportion of short reads mapped to assembled contigs. (PDF 70 kb) Additional file 8: Figure S5. Completeness assessment of transcriptome assemblies by CEGMA and BUSCO referring to CVG. (PDF 468 kb) Additional file 9: Table S4. Completeness assessment of comprehensive transcript sequence sets of bony vertebrates based on numbers of detected genes. (PDF 79 kb)

## Abbreviations

CEGMA: Core Eukaryotic Genes Mapping Approach
CEG: Core Eukaryotic Genes
CVG: Core Vertebrate Genes
dpo: days post oviposition
G6PD: Glucose-6-phosphate dehydrogenase
H6PD: Hexose-6-phosphate dehydrogenase
nt: Nucleotides
aa: Amino acids
PCR: Polymerase chain reaction
PE: Paired-end
