# Supplementary material for: Optimizing and benchmarking de novo transcriptome sequencing: from library preparation to assembly evaluation
Source: BMC Genomics. 2015 Nov 18;16:977. doi: 10.1186/s12864-015-2007-1 (PMC4652379; doi:10.1186/s12864-015-2007-1)
Supplement: Additional file 1: Table S1. — Detailed assembly statistics based on numbers of detected genes. (PDF 61 kb) [file 12864_2015_2007_MOESM1_ESM.pdf]

Additional file 1: Table S1. Detailed assembly statistics.

Assembly No.	Number of fragments (×10^6)		Original assembly		Assembly filtered by mapping count (≥5)		N50 length (bp)		Median length (bp)	
	Raw	After QC	Number of contigs	Number of subcomponents	Number of contigs	Number of subcomponents	All contigs	Longest isoform	All contigs	Longest isoform
1	30.986	22.719	222178	168924	106323	62636	3091	1891	984	632
2	33.683	21.224	228165	159338	94371	45267	3634	2248	1640	1007
3	4.757	3.569	104985	83417	37504	22331	3093	2573	1706	1362
4	32.151	23.712	417291	291424	204328	104294	3693	1389	1045	574
5	25.099	16.037	383737	246347	149926	56669	4149	2124	1956	924
6	91.361	75.929	798982	562528	358433	182611	3956	1216	1122	697
7	94.439	82.453	787608	541906	375297	191055	3860	1199	1100	687
8	97.537	81.033	525154	348570	250433	115476	4090	1378	1307	722
9	NA	326.676	1214573	852257	653132	387456	2680	1086	848	671
10	NA	326.676	1087900	745363	748019	422329	4854	3067	1723	1060
11	NA	39.593	1465425	721986	972512	330937	3755	1208	1497	684
12	NA	33.251	1464412	741241	945799	314023	2898	1188	1256	693
13	NA	326.676	1562282	939252	996336	457323	3897	1338	1132	640

Details of these assemblies are described in Tables 1 and 2.
