# Supplementary material for: Optimizing and benchmarking de novo transcriptome sequencing: from library preparation to assembly evaluation
Source: BMC Genomics. 2015 Nov 18;16:977. doi: 10.1186/s12864-015-2007-1 (PMC4652379; doi:10.1186/s12864-015-2007-1)
Supplement: Additional file 2: Figure S1. — Size distribution of contigs in assemblies. (PDF 80 kb) [file 12864_2015_2007_MOESM2_ESM.pdf]

Additional file 2

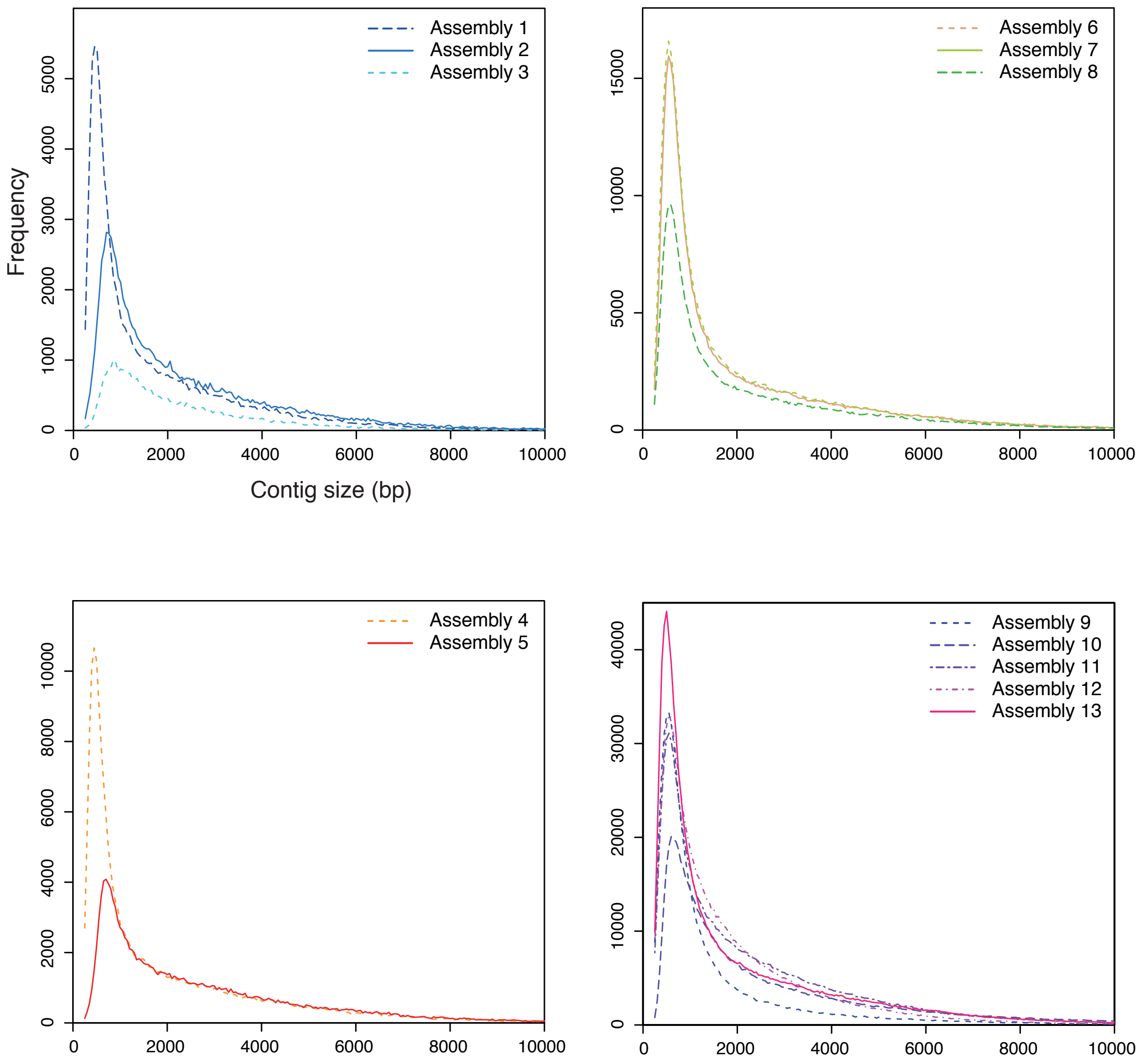

Figure S1. Size distribution of contigs in assemblies.

For individual assemblies, the contig size distributions are grouped by embryonic RNA sources. Also, the size distributions of the contigs of the integrated assemblies are shown.
