# Supplementary material for: Optimizing and benchmarking de novo transcriptome sequencing: from library preparation to assembly evaluation
Source: BMC Genomics. 2015 Nov 18;16:977. doi: 10.1186/s12864-015-2007-1 (PMC4652379; doi:10.1186/s12864-015-2007-1)
Supplement: Additional file 3: Figure S2. — Size distribution of prepared and sequenced fragments. (PDF 121 kb) [file 12864_2015_2007_MOESM3_ESM.pdf]

Additional file 3

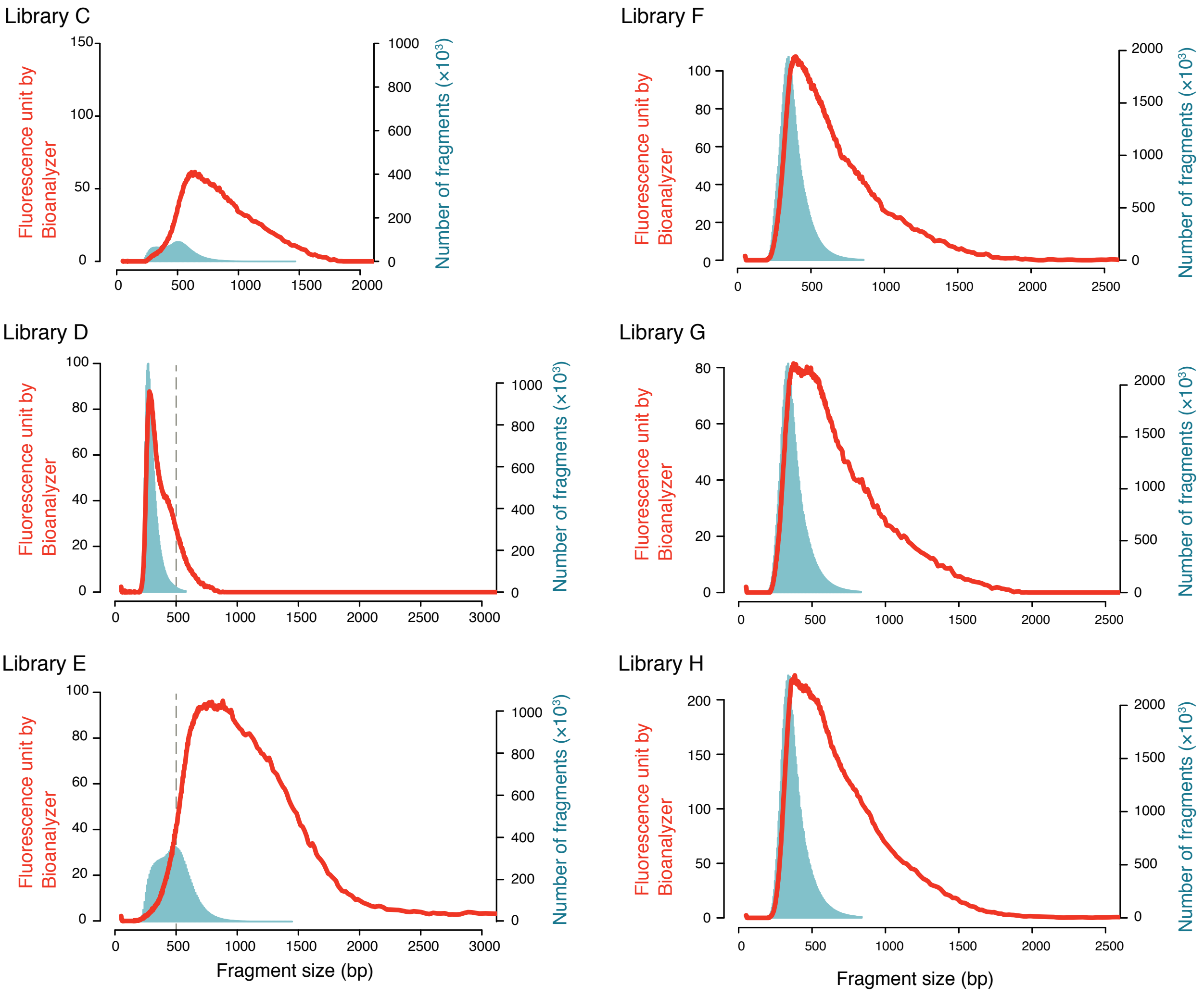

Figure S2. Size distribution of prepared and sequenced fragments.

Fragment size distributions are shown for Library C, which was prepared with the same protocol to Library B and sequenced with MiSeq; Library D and Library E derived from the 9 dpo embryo; Library F, Library G, and Library H from 30 dpo embryo. See Table 1 and the legend of Figure 2 for details.
