# Supplementary material for: Optimizing and benchmarking de novo transcriptome sequencing: from library preparation to assembly evaluation
Source: BMC Genomics. 2015 Nov 18;16:977. doi: 10.1186/s12864-015-2007-1 (PMC4652379; doi:10.1186/s12864-015-2007-1)
Supplement: Additional file 4: Figure S3. — Paralogs in the CEGs. (PDF 95 kb) [file 12864_2015_2007_MOESM4_ESM.pdf]

Additional file 4

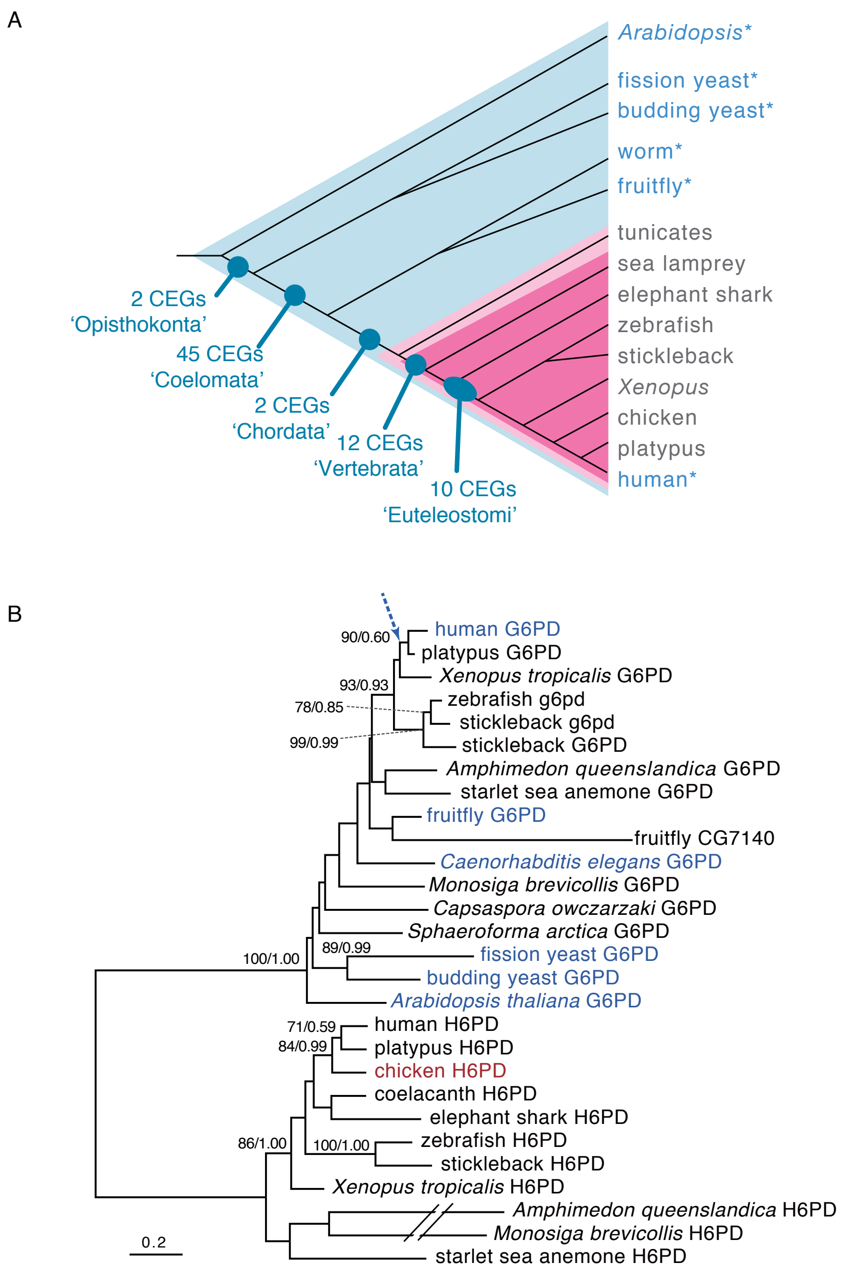

Figure S3. Paralogs in the CEGs

(**A**) Duplication events in CEGs in the lineages leading to vertebrates and early

vertebrates. Based on the gene trees provided by Ensembl, 71 duplication events were

observed in 64 CEGs. The taxon names were given by Ensembl. (**B**) Molecular phylogenetic tree of G6PD/H6PD genes inferred in a maximum-likelihood framework based on 239 amino acid sites unambiguously aligned. This phylogeny shows that the chicken gene identified by CEGMA as a member of KOG0563 (red) is not orthologous to G6PD (blue) but is included in the group of H6PD genes, which duplicated before the split between the choanoflagellate and metazoan lineages. A blue arrowhead indicates the branch from which a chicken G6PD ortholog would be splitting off. Bootstrap probabilities over 70 in the maximum-likelihood analysis and posterior probabilities based on a Bayesian framework are shown at the nodes, in order.
