# Supplementary material for: Optimizing and benchmarking de novo transcriptome sequencing: from library preparation to assembly evaluation
Source: BMC Genomics. 2015 Nov 18;16:977. doi: 10.1186/s12864-015-2007-1 (PMC4652379; doi:10.1186/s12864-015-2007-1)
Supplement: Additional file 6: Table S3. — Completeness scores of transcriptome assemblies based on numbers of detected genes. (PDF 62 kb) [file 12864_2015_2007_MOESM6_ESM.pdf]

Additional file 6: Table S3. Completeness scores of transcriptome assemblies based on numbers of detected genes

Assembly No.	CEGMA referring to CEG				CEGMA referring to CVG				BUSCO referring to CVG				BUSCO referring to the vertebrate set			
	Complete		Partial		Complete		Partial		Complete		Partial		Complete		Partial	
	Number of genes	Proportion of genes	Number of genes	Proportion of genes	Number of genes	Proportion of genes	Number of genes	Proportion of genes	Number of genes	Proportion of genes	Number of genes	Proportion of genes	Number of genes	Proportion of genes	Number of genes	Proportion of genes
1	241	0.972	243	0.980	216	0.927	229	0.983	223	0.957	231	0.991	2450	0.810	2613	0.864
2	241	0.972	245	0.988	221	0.949	231	0.991	224	0.961	231	0.991	2470	0.817	2648	0.876
3	237	0.956	246	0.992	201	0.863	220	0.944	214	0.919	226	0.970	2166	0.717	2354	0.779
4	247	0.996	247	0.996	216	0.927	227	0.974	227	0.974	230	0.987	2541	0.841	2713	0.897
5	239	0.964	245	0.988	218	0.936	227	0.974	225	0.966	228	0.979	2557	0.846	2704	0.894
6	240	0.968	247	0.996	217	0.931	228	0.979	226	0.970	231	0.991	2682	0.887	2842	0.940
7	245	0.988	247	0.996	215	0.923	225	0.966	226	0.970	229	0.983	2658	0.879	2811	0.930
8	246	0.992	248	1.000	215	0.923	226	0.970	225	0.966	231	0.991	2526	0.836	2687	0.889
9	229	0.923	247	0.996	217	0.931	228	0.979	230	0.987	232	0.996	2698	0.892	2861	0.946
10	246	0.992	247	0.996	219	0.940	232	0.996	224	0.961	232	0.996	2790	0.923	2910	0.963
11	219	0.883	248	1.000	184	0.790	231	0.991	203	0.871	230	0.987	2425	0.802	2783	0.921
12	194	0.782	248	1.000	144	0.618	220	0.944	175	0.751	226	0.970	2164	0.716	2667	0.882
13	247	0.996	248	1.000	231	0.991	232	0.996	233	1.000	233	1.000	2884	0.954	2954	0.977

Details of these assemblies are described in Tables 1 and 2.
