# Supplementary material for: Optimizing and benchmarking de novo transcriptome sequencing: from library preparation to assembly evaluation
Source: BMC Genomics. 2015 Nov 18;16:977. doi: 10.1186/s12864-015-2007-1 (PMC4652379; doi:10.1186/s12864-015-2007-1)
Supplement: Additional file 7: Figure S4. — Proportion of short reads mapped to assembled contigs. (PDF 70 kb) [file 12864_2015_2007_MOESM7_ESM.pdf]

Additional file 7

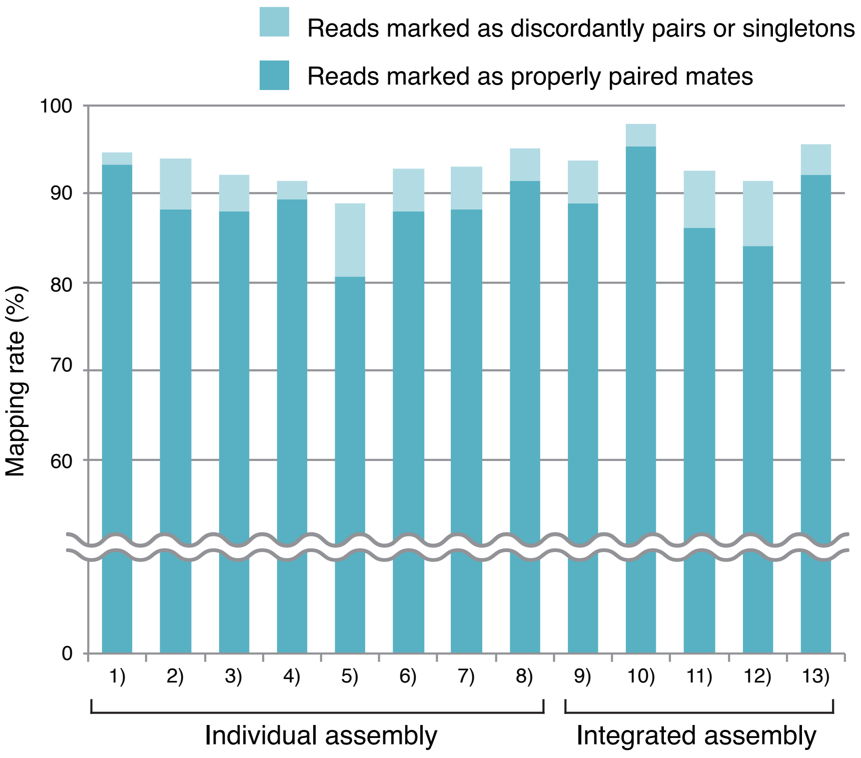

Figure S4. Proportion of short reads mapped to assembled contigs.

Sequence reads were mapped to the assembled contigs by bowtie2. Mapping properties of sequence reads were indicated by the SAM flags from the mapping results. Details of the assemblies are included in Table 2 and Additional file 4.
