# Supplementary material for: Optimizing and benchmarking de novo transcriptome sequencing: from library preparation to assembly evaluation
Source: BMC Genomics. 2015 Nov 18;16:977. doi: 10.1186/s12864-015-2007-1 (PMC4652379; doi:10.1186/s12864-015-2007-1)
Supplement: Additional file 8: Figure S5. — Completeness assessment of transcriptome assemblies by CEGMA and BUSCO referring to CVG. (PDF 468 kb) [file 12864_2015_2007_MOESM8_ESM.pdf]

Additional file 8

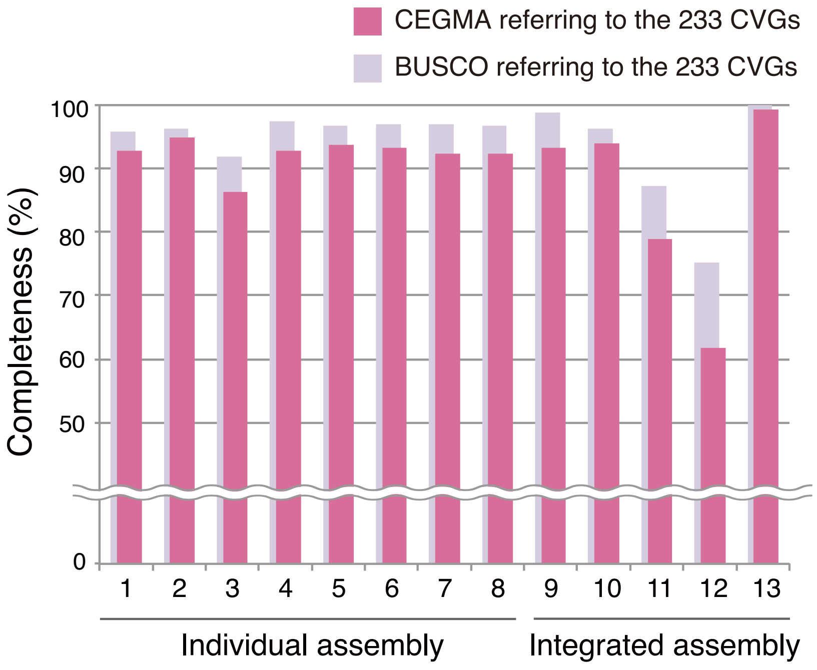

Figure S5. Completeness assessment of transcriptome assemblies by CEGMA and BUSCO referring to CVG.

The scores indicate proportions of the genes recognized as ‘complete’ in individual assemblies by CEGMA and BUSCO in the 233 CVGs. The scores are highly correlated between CEGMA and BUSCO (R=0.99; p=2.3×10^{-10}).
