# Supplementary material for: Optimizing and benchmarking de novo transcriptome sequencing: from library preparation to assembly evaluation
Source: BMC Genomics. 2015 Nov 18;16:977. doi: 10.1186/s12864-015-2007-1 (PMC4652379; doi:10.1186/s12864-015-2007-1)
Supplement: Additional file 9: Table S4. — Completeness assessment of comprehensive transcript sequence sets of bony vertebrates based on numbers of detected genes. (PDF 79 kb) [file 12864_2015_2007_MOESM9_ESM.pdf]

Additional file 9: Table S4. Completeness assessment of comprehensive transcript sequence sets of bony vertebrates based on numbers of detected genes

Species[1]	CEGMA referring to CEG				CEGMA referring to CVG				BUSCO referring to CVG[2]				BUSCO referring to the vertebrate set[2]			
	Complete		Partial		Complete		Partial		Complete		Partial		Complete		Partial	
	Number of genes	Proportion of genes	Number of genes	Proportion of genes	Number of genes	Proportion of genes	Number of genes	Proportion of genes	Number of genes	Proportion of genes	Number of genes	Proportion of genes	Number of genes	Proportion of genes	Number of genes	Proportion of genes
Pan troglodytes	230	0.9274	243	0.9798	220	0.9442	225	0.9657	222	0.953	225	0.966	2778	0.919	2818	0.932
Pongo abelii	227	0.9153	246	0.9919	217	0.9313	230	0.9871	225	0.966	228	0.979	2754	0.911	2848	0.942
Macaca mulatta	223	0.8992	240	0.9677	202	0.867	224	0.9614	219	0.940	226	0.970	2761	0.913	2907	0.962
Anas platyrhynchos	159	0.6411	200	0.8065	185	0.794	226	0.970	216	0.927	229	0.983	2304	0.762	2505	0.829
Meleagris gallopavo	161	0.6492	209	0.8427	181	0.7768	225	0.9657	221	0.948	228	0.979	2275	0.753	2463	0.815
Lepisosteus oculatus	210	0.8468	232	0.9355	221	0.9485	231	0.9914	228	0.979	232	0.996	2639	0.873	2714	0.898
Astyanax mexicanus	214	0.8629	239	0.9637	197	0.8455	221	0.9485	206	0.884	221	0.948	2434	0.805	2576	0.852
Takifugu rubripes	204	0.8226	244	0.9839	193	0.8283	218	0.9356	217	0.931	224	0.961	2616	0.865	2717	0.899

[1] Annotated transcript sequences were obtained from Ensembl version 75

[2] BUSCO run employed the 'transcriptome' mode.
